# LGBTQI + Migrants: A Systematic Review and Conceptual Framework of Health, Safety and Wellbeing during Migration

**DOI:** 10.3390/ijerph19020869

**Published:** 2022-01-13

**Authors:** Vanessa Yarwood, Francesco Checchi, Karen Lau, Cathy Zimmerman

**Affiliations:** Department of Global Health and Development, Faculty of Public Health and Policy, London School of Hygiene and Tropical Medicine, London WC1E 7HT, UK; francesco.checchi@lshtm.ac.uk (F.C.); karen524@connect.hku.hk (K.L.); cathy.zimmerman@lshtm.ac.uk (C.Z.)

**Keywords:** forced migration, health, protection, diverse SOGIE, LGBTQI+, systematic review

## Abstract

The health and safety of LGBTQI+ migrants or migrants who are of diverse sexual orientation, gender identity or expression (SOGIE) remains an under-studied area, particularly for the period during transit from their place of origin to destination. This systematic review aims to describe the literature on the health risks and consequences among SOGIE migrants during transit and examine their access and use of services. Six peer-reviewed databases and websites of nine large migration organisations were searched to identify the literature on forced migrants and sexual and gender minorities. Twenty English-language studies from 2000–2021 were included and analysed drawing on a conceptual framework. Studies emerged from six regions and the majority of research participants identified as gay men. In general, quality appraisal demonstrated studies as either medium or high quality. Findings suggested five common themes associated with SOGIE health and well-being, including: daily exposure to discrimination, harassment and violence; coping, social support and resilience; access to services; mental health; and physical and sexual health. Depression, anxiety and post-traumatic stress disorder (PTSD) were prevalent amongst SOGIE migrants, particularly when associated with detention or camp environments, and were exacerbated by social isolation. Barriers to accessing healthcare were identified and specific sexual health services were often found lacking, especially for trans persons. Unsurprisingly, during transit, SOGIE migrants are very likely to experience the double marginalisation of their migrant or minority status and their gender identity. Results indicate that services for SOGIE migrants need to tailor service access and support approaches to respond to the particular health and protection needs of SOGIE individuals in each setting.

## 1. Introduction

With 82.4 million forcibly displaced persons worldwide at the end of 2020, and increasing numbers of refugees and asylum seekers registered each subsequent year, migration is a feature of the modern world that is set to continue [1]. Individuals with diverse sexual orientation, gender identity or expression (SOGIE) experience persecution in many countries, with subsequent health and safety risks well reported, including physical and sexual violence, abuse and discrimination on a daily basis including from institutions, and resulting concealment of identity [2,3]. In addition many states criminalise those who identify or express sexual and gender diversity [4]. As a result, displacement due to diverse SOGIE is a recognised cause to seek asylum [5,6,7,8], with the creation of normative documents such as the Yogyakarta Principles to define and apply human rights values to these individuals [9,10]. Additionally, there are many SOGIE individuals who among migrant populations who are forced to leave their countries because of conflict, natural disasters, humanitarian crises and diminished resources due to climate change. Challenges related to displacement of refugees and asylum seekers with diverse SOGIE from their country of origin, as well as integration and resettlement into Western countries have been well described [11,12,13,14,15,16,17], but less is known about experiences along transit routes. Terminology for individuals with diverse sex, gender and sexuality varies in the literature and in practice, and often includes LGBTQI+ and gender non-conforming individuals. In this paper we use the terminology diverse SOGIE to remain inclusive and capture the limited literature on this subject.

The transit phase of migration is a complex period when individuals move away from their place of origin to a safe destination [18]. For many, transit is non-linear or even circular, and often transit points can merge into the final destination [19,20,21,22]. Countries can serve as transit for some individuals and destination for others, with transit zones developing based on proximity and opportunity to cross borders, and irregular movement across borders commonplace [23]. Individuals who migrate may not access official migration services or resettlement pathways immediately, or only claim asylum once a final, not interim, destination has been reached. Transit is often a dangerous time with precarious travel across challenging terrain, with exposure to smugglers, traffickers or armed groups, and which can often involve arbitrary detention, encampment, or homelessness [24]. For many individuals, particularly marginalised groups, travel from origin to destination can involve abuse, exploitation, sexual violence, and in some cases, kidnapping and torture [25]. Prolonged transit periods can negatively impact individual’s physical and mental health. Similarly, access to healthcare is often limited [21]. At the time of publication over 3500 people had been recorded as missing in transit across the world in 2021 [26]; however, the true number is likely to be significantly higher, and many more are likely to be subjected to torture and violence. The past decade has seen increasing barriers to migration flows through securitisation, forced encampment, and policies to externalise borders or criminalise those attempting to cross borders irregularly [19]. These restrictions increase the risk of exploitation of those forced to migrate by smugglers offering passage.

Compared to general migrant populations, migrants with diverse SOGIE are often at additional risk of abuse, discrimination or reduced access to services during transit because of their identity, both from cisgender, heteronormative forced migrants, and authorities or locals, particularly if their identity is inadvertently revealed [11,27,28]. Minors with diverse SOGIE may also face additional vulnerabilities. The small body of literature on SOGIE forced migrants suggest that SOGIE individuals are likely to require tailored services for their reproductive health, mental health, or HIV care needs, and special housing support [29,30]. There is evidence to suggest that persons with diverse SOGIE who have been forced to leave their homes may already suffer symptoms of post-traumatic stress disorder (PTSD), and may have higher prevalence of mental illness compared to non-diverse SOGIE forced migrants [31,32]. Although not sufficiently studied, reports suggests that the needs of mobile SOGIE groups are not managed holistically by service providers and humanitarian organisations [33]. However, through our search, we identified several sets of guidance for service operations [30,34,35,36].

**Figure 1:**
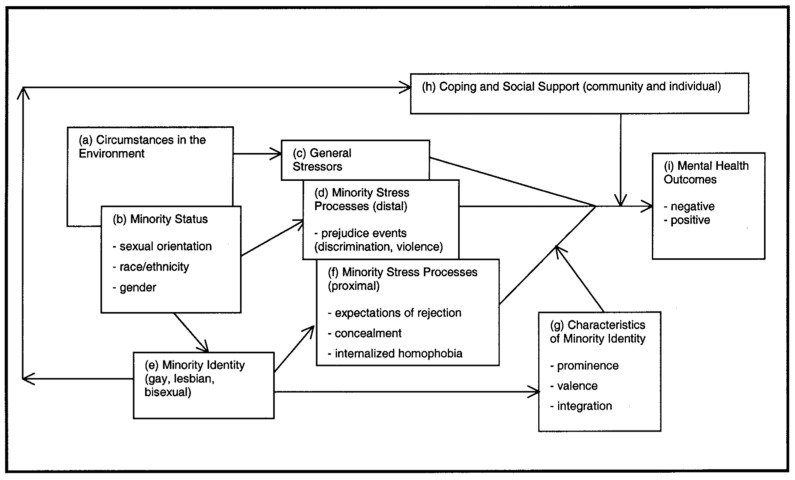
Minority stress model [39].

Emerging discourse suggests that addressing forced migration for those with diverse SOGIE requires an intersectional approach to consider the interaction between factors such as age, disability, ethnicity and socio-economic status that might compound marginalisation associated with their minority identity [37,38]. The minority stress model (Figure 1) can be used as a framing tool to describe, for example, excess stress experienced by individuals based on their minority status [39]. Minority status reflects intersections of gender, sexual orientation, race, and status as a forced migrant. The model highlights how these individualities affect how external or internal stress is perceived and experienced, and subsequently how this goes on to affect mental health outcomes. Coping and social support at individual and community levels is seen as an important mediating factor on health outcomes.

To date, studies on the risk and protective factors and health consequences among forced migrants with diverse SOGIE during their migration journey are limited, and there are even fewer studies evaluating interventions. This review responds to these knowledge gaps by assessing current evidence on SOGIE migrants. [40,41].

## 2. Materials and Methods

The aim of this review was to identify and analyse the literature on the health risks, consequences, and access to services amongst forced migrants with diverse SOGIE to inform future policy and programmatic responses for SOGIE populations. Specifically, the study objectives were to examine health risks and protective factors, document mental and physical health consequences, and examine health and other support needs during migration including description of services. The study was registered in PROSPERO (registration number: CRD42021241418).

Table 1 shows the inclusion and exclusion criteria using the SPIDER tool (sample, phenomenon of interest, study design, evaluation and research type) [42,43].

We use the term individuals with diverse SOGIE to represent those who do not follow the traditional Western normative binary gender norms or sex assigned at birth [33]. We used the term ‘forced migrant’ to include refugees, asylum seekers, and other forced irregular migrants, to capture individuals on migration routes before protection status is determined. Voluntary migrants were not included in the review.

The transit phase of the migration journey includes individuals who have recently undertaken or are undertaking migration. Each eligible study was reviewed for data collected on experiences during transit or shortly after, including those in temporary locations such as detention centers or holding camps. Data collected on migrants’ experiences in destination countries were limited to the impact of the migration journey and did not include long-term assimilation challenges. Where there was ambiguity, information was reviewed by team members. Information on pre-departure services was not collected. The major outcomes of interest were health and safety of forced migrants, and access to services during or immediately after transit.

Methods follow the reporting structure set out by Preferred Reporting Items for Systematic Reviews and Meta-Analyses (PRISMA) guidelines [44]. The peer reviewed literature was searched within CINAHL Plus, EMBASE, MEDLINE, PsycINFO, Pubmed, Scopus databases, and included title, abstract, keywords and subject headings. The review used advanced searches of two concepts encompassing forced migrants and diverse SOGIE, and used MeSH terms where relevant. The following strategy was used for Ovid databases as an example:

Key term 1: MH “refugees” or MH “undocumented immigrants”
  or refugee* or asylum seeker* or undocumented migra* or forced migra* or irregular migra* or displace*;
Key term 2: MH “sexual and gender minorities”
  or gender non-conform* or gender minorit* or gay or lesbian* or LGBT* or queer* or bisexual* or intersex or SOGIE* or transsex* or transgend* or trans peop* or non-binary or homosex*;

Limit to y = “2000 − Current”.

N.B. asterisk is used as truncation

The grey literature comprised information provided from the UNHCR Global Roundtable on Protection and Solutions for Lesbian, Gay, Bisexual, Transgender, Intersex, Queer (LGBTIQ+) People in Forced Displacement, and publications of key migration organisations: United Nations High Commissioner for Refugees (UNHCR), International Organisation for Migration (IOM), World Health Organisation (WHO), The Edge Effect, Women’s Refugee Commission (WRC), Organisation for Refuge, Asylum and Migration (ORAM), Human Rights Watch (HRW), Amnesty International, and the Office of the United Nations High Commissioner for Human Rights (OHCHR). Websites were searched using the terms listed above, or through hand-searching. Bibliographies of eligible articles were also hand-searched.

**Figure 2:**
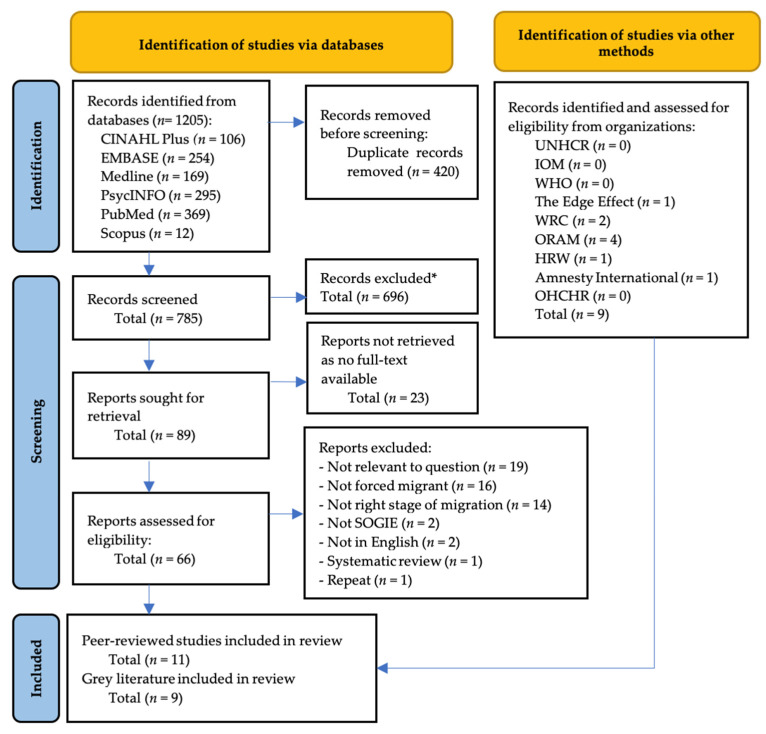
Adapted PRISMA 2020 flow diagram for new systematic reviews, including searches of databases, registers and other sources. N.B. asterisk is used as truncation.

Articles were downloaded onto *Mendeley Desktop*, version 1.19.8, 2008∓2020 Mendeley Ltd., and underwent de-duplication, screening, retrieval of full-text, and assessment for eligibility. Queries were discussed with other team members. Where findings were reported in more than one study, the report was included that provided the most information, this applied to one study. The total yield was 1205, after assessment just 20 studies were included. The study selection process and reasons for exclusion can be found in Figure 2.

Data were extracted and recorded in Microsoft Excel. Study information extracted included the author, journal, objectives, setting, definitions of both SOGIE term used and forced migrant status, study design, methods and sampling, and study population. For qualitative studies, data were extracted for each study by recording the main thematic findings. For quantitative data individual findings were recorded for each study as appropriate.

Quality appraisal of the evidence was evaluated using the Critical Appraisal Skills Programme (CASP) tool for qualitative studies [45], and the Appraisal of Cross-Sectional Studies (AXIS) tool for cross-sectional survey studies [46], or both for mixed-methods. Each study was graded into scores of high (over 66%), medium (33–66%) or low (less than 33%). A second reviewer was involved in grading of quality assessments.

For qualitative studies, after familiarisation with the data, line by line coding was conducted to extract information through an inductive approach and ensure themes were grounded in the data. Codes were compared across studies and common themes identified and synthesised. Quantitative data were analysed individually, and meta-analysis was not possible due to heterogeneity of methods and results. The quantitative results informed the qualitative themes.

During conception of the review the minority stress model was used as a framing tool to examine the relationship between minority status and stressors, and their impact on mental health outcomes [39]. After completing the review analysis, we developed a new framework drawing on the minority stress model to describe pathways to mental and physical health outcomes seen in forced migrants with diverse SOGIE. We defined exposures and mediators that influenced these pathways and reflected how intersecting minority identities shaped outcomes.

## 3. Results

### 3.1. Study Characteristics

**Figure 3:**
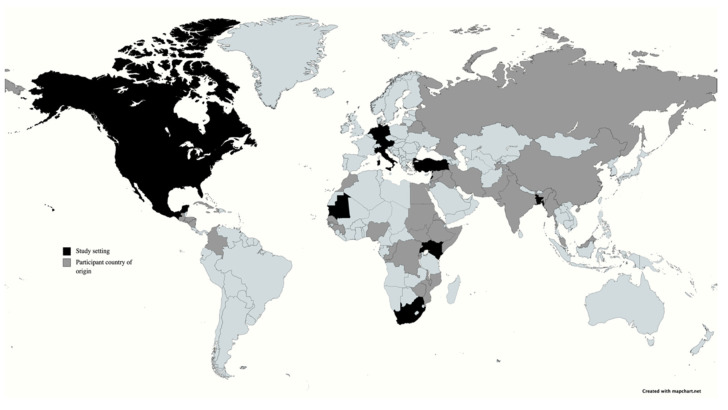
World map representing setting of studies (black) and country of origin of participants (grey).

Twenty studies were included in the final sample, of which fifteen were qualitative, four were quantitative, and one mixed-methods. Figure 3 depicts the locations where studies were conducted globally, and where study participants originated from. The majority of studies (fourteen) took place in Europe, Africa and the Middle East, whilst study participants originated from over 48 countries. The majority of studies involved participants in the urban environment, two took place in camp settings [33,47].

### 3.2. Gender Terminology

Studies used varying gender terms, with eight studies adopting umbrella terms such as ‘queer’ [48,49,50], ‘diverse SOGIESC’ [51,52], or a form of ‘LGBTQ’ [47,53,54]. Others provided a breakdown of numbers of participants within gender categories according to either self-identification or categories provided by the authors. The vast majority of participants in both qualitative and quantitative studies who detailed gender categories identified as gay or men who have sex with men (MSM) (n = 542), followed by transgender women (n = 86), lesbian (n = 76), bisexual (n = 54), non-binary (n = 52), and transgender men (n = 23). All studies used non-random sampling of diverse SOGIE populations, therefore individuals had to be associated with an LGBTQ organisation or sample population to be included. Table 2 shows detailed study characteristics.

The majority of studies evaluated by quality assessment tools were graded as medium quality (ten), eight were high quality, and two low were quality. Although qualitative studies reported on appropriate research design and methodology, they often did not record adequate details on recruitment of participants, how data were collected and how analysis was performed, which was true of both the peer-reviewed and grey literature. The majority of qualitative studies did not comment on the relationship, influence and resultant bias between the researchers and participants. Quantitative studies tended to be missing information on non-responders and often did not justify sample sizes.

### 3.3. Summary of Findings

The inductive coding approach identified common findings that were synthesised into five thematic areas:

- Daily exposure to discrimination, harassment and violence;
- Coping, social support and resilience;
- Access to services;
- Mental health; and
- Physical and sexual health

#### 3.3.1. Daily Exposure to Discrimination, Harassment and Violence

Ten studies reported that other refugees and migrants subjecting participants to abuse or discrimination related to their diverse SOGIE status, who were from the same or different nationality. Discrimination occurred in encamped settings in Europe and Bangladesh, in urban environments in South Africa, Turkey or Uganda, or both in Kenya. Participants reported danger associated with being housed with compatriots [63], fearing sexual assault, social isolation, and these fears or abuses were the cause of suicidal thoughts for some [64]. Authorities were also a source of significant harassment and abuse. In Europe and South Africa, immigration officials were reported as perpetrators, whereas in other narratives, the police were heavily implicated. This was the case with gay men in Mauritania experiencing sexual assault, and in three Mexican studies reporting insults relating to SOGIE on the street and theft of money and belongings. In East Africa forced migrants experienced abusive behavior when they were arrested in Kenya, and reported frequent threats and in one case sexual assault by police in Uganda [56]. Abuse and discrimination at the hands of local residents were also reported in twelve studies, including in South Africa, Turkey, and on buses in Mexico and in Mauritania. In Mexico, migrants feared trans-femicide due to reports of hate crimes committed to trans people in the region. Sexual violence and assault were particular features reported in Uganda, Kenya, and in Central American countries [50,52]. In several studies the intersection between SOGIE status and race was discussed and related to abuse from locals. Individuals emphasised the compounded effect of being both a refugee and a sexual minority, with significant xenophobia in South Africa, and racism in Italy and Germany [17]. Participants in several studies protected themselves from harassment by hiding their identity or sexual orientation. Concealment was practiced globally, in European countries, to Turkey, Uganda, Mauritania, Libya, South Africa and Mexico. Two studies reported instances in which violence was not compounded by individual SOGIE status, such as during sea transit from Turkey to Greece, and in detention camps in Libya where extreme violence was experienced by all. It is likely that individuals’ SOGIE status was not revealed in these cases. Many studies included quotes indicating that individuals understood not to reveal their sexual orientation: *“Somebody gave the advice just not say you’re gay”* [63].

Accommodation issues were raised in numerous narratives, with individuals often citing not feeling safe in accommodation. In Cape Town, individuals reported theft of belongings and trouble from substance misusers. In Turkey and Kenya, individuals experienced discrimination from private landlords, and some shelters in Mexico did not allow trans people to take hormone therapy. Findings indicated that individuals commonly experienced lack of stable housing and were constantly having to move, and others had little autonomy over decisions related to shelter. Studies that reported on employment highlighted difficulties individuals faced both when trying to obtain and maintain work. Reports of discrimination related to SOGIE status when looking for work in Mexico, South Africa and Turkey is explained by a participant in Turkey: *“When you enter a shop, they look at you like you’re a creature. When you ask for job, they say yes we need someone, but not you”* [64]. Even when in work, employment could be jeopardised if SOGIE status was revealed.

Two quantitative studies reported specifically on discrimination. One study in Lebanon reported that 30% of recently migrated Syrian men experienced discrimination for being gay compared to 6% recently migrated Iraqi men or 8% Lebanese-born Palestinians (*p* < 0.001) [59]. Another study in Lebanon found higher levels of sexual minority discrimination (*p* < 0.01) and sexual minority assault (*p* < 0.001) among displaced Syrians when compared to the non-displaced Lebanese population [57]. Several quantitative studies reported on employment and income. A study on LGBTQ asylum seekers in Canada found that 72.4% of participants were employed, and in Lebanon the proportion of MSM refugees that were working was similar [58,59]. Two Lebanese studies reported that displaced participants received lower income compared to participants who hadn’t been displaced [57,59].

#### 3.3.2. Coping, Social Support and Resilience

Feelings of social isolation and loneliness were discussed in many studies in the review. These feelings appeared to be exacerbated by obstruction to participation in community or religious structures because of one’s SOGIE status. Findings indicated that in some instances, individuals were excluded from participation in groups when SOGIE status was revealed or discovered [63]. Individuals held back revealing their SOGIE status to peers, despite wishing to be able to express their identity. Loneliness and isolation were also exacerbated by the physical location in which individuals were based, for example by resettlement in traditional satellite cities in Turkey or rural camps in Germany.

One quantitative study looked at social isolation of LGBTQ asylum seekers in Canada using two validated tools. In both tools, participants scored low for social and emotional support when compared to US population norms. In this study, the most common support was from significant others (41.2%) and LGBTQ friends via the internet (35.4%), with least support from family (13.3%) and religious communities (5.8%) [58]. It is important to note that this study included those who had been in Canada for very short periods of time but also included those who had been residing there for over 10 years.

Several studies commented on protective features. In particular, the use of digital media was seen as a way to access social support from loved ones at home, make new contacts, or learn about asylum procedures and services available. This form of communication was a source of strength and support. In Kenya, forced migrants set up an online media support page to highlight their problems, which received international support in the form of advocacy and money for medication and transport [47]. Some individuals who engaged with online activism in Uganda had a sense of agency as well as connection to others in a similar situation [54].

#### 3.3.3. Access to Services

Reporting on services was variable, with some of the grey literature providing detailed examples of good practice undertaken by organisations. Other articles focused more on whether participants could access assistance. Although access to medical care and mental health and psychosocial support was reported for forced migrants with diverse SOGIE in some locations, others faced barriers and challenges to find and use services. Individuals were refused treatment or discriminated against by medical staff in refugee camps in Bangladesh, Kenya, Mexico and transgender women were denied hormonal treatment in South Africa. In Uganda, mistrust between participants and medical staff was raised as well as the inability to access medical care after sexual assault or pregnancy resulting from assault. Some specific services for those with diverse SOGIE were lacking, such as testing and treatment for STIs and HIV, and hormone therapy for trans persons in Kenya. Sometimes access to services was dependent on refugee or asylum status, such as receiving free HIV care in Mauritania. In Kenya, individuals reported that they feared deportation if they used sexual assault services, and others reported that specific services for those with diverse SOGIE were lacking. Access to social support services was difficult for trans persons in Kenya, who reported rejection from women’s organisations [52]. Physical distances also posed challenges for those living in satellite locations, such as in Turkey, where services were based in major cities [50]. In Bangladesh, participants lamented the lack of a safe zone such as those that were available for cisgender women [33].

Two quantitative studies specifically reported on healthcare access or utilisation. In Germany, residence in the LGBTIQ shelter was associated with a higher probability in accessing ambulatory care (*p* = 0.04), after controlling for chronic illness and subjective health. Similarly, for mental health, residing in an LGBTIQ shelter was associated with a higher probability of obtaining mental health care, when controlling for demand (*p* < 0.001) [53]. In Lebanon, around 30% of all study participants reported seeing a doctor in the past 12 months, and this was similar between displaced and non-displaced populations [59]. A study in Canada highlighted that 39% of participants said that cost had prevented them from accessing mental healthcare since arriving in North America. However, the study found that 70.45% of those screening positive for mental distress on the Refugee Health Screener 15 (RHS15) were seeing a mental health counsellor [58].

#### 3.3.4. Mental Health

Mental health was mentioned in nine of the qualitative studies, with some individuals referencing sexual assault as traumatising, causing nightmares and feelings of stress. Nightmares and anxiety symptoms were also experienced by refugees in Turkey, and self-isolation and fear of being out led to depression and thoughts of suicide in Uganda and Turkey. One study looked at how detention in Mexico and US exacerbated mental health, especially when trans women were held in male facilities, and inappropriate placement of a trans woman in a male camp also occurred in Europe. Two studies referenced worsening mental health and anxiety of participants after being returned or deported to countries of origin. Anxiety was discussed in relation to being stuck in limbo and waiting for asylum or resettlement decisions. In Turkey and Mauritania, in particular, this sense of limbo was linked to anxiety and depression. Others found waiting created boredom and a feeling of being trapped. Poor mental health was also experienced in destination countries in Europe.

Some individuals expressed a sense of freedom and hope for the future on arrival to destination countries, however others said they felt hopeless about their current situation and perceived that life was better prior to migration than what they were experiencing now [17]. Conflicting feelings were reported within some studies, as individuals suggested that although they no longer feared for their lives, their daily quality of life had reduced [50].

Five studies reported quantitative results related to mental health, using different measurement tools. Studies in Europe and in Lebanon measured prevalence of a provisional diagnosis of PTSD using either the PCL5 or the PCL-C. For those in Europe, 64.9% of participants met the provisional criteria for PTSD, whereas for displaced Syrians in Lebanon 33% met the PTSD cut-off (significantly higher than the non-displaced Lebanese comparative group, *p* < 0.001) [55,57]. These two studies cannot be directly compared as the study populations and settings are very different. One of these studies reported that the most distressing event for participants occurred prior to migration (64.9%), with 10.8% stating the most distressing event occurred during travel [55].

Two studies, in Lebanon and Germany, examined prevalence of depression and anxiety when compared to other populations. Higher proportions of depression and anxiety were seen in the displaced population in the Lebanese study compared to the non-displaced group (depression 63% vs. 43.8%, *p* < 0.001; severe anxiety 21.3% vs. 13.1%, *p* < 0.05), and a higher proportion of the LGBTIQ population screened positive for depression and/or anxiety in the German study when compared to non-LGBTIQ group (70% vs. 34%, OR 4.5 [1.79–12.47], *p* < 0.001) [53,57]. Being displaced was also associated with depression and PTSD on linear regression models in Lebanon when adjusting for sociodemographic characteristics (*p* < 0.05 depression, *p* < 0.001 PTSD).

One study in Canada measured mental distress through the RHS15 tool, with 80.2% participants screening positive. On multivariable linear regression modelling, factors positively associated with mental distress included identity disclosure and loneliness (*p* < 0.05 and *p* < 0.001), while proficiency in English and being granted asylum were protective (*p* < 0.05 and *p* < 0.007) [58].

#### 3.3.5. Physical and Sexual Health

Many forced migrants with diverse SOGIE described engaging in sex work at points during their migration journey. In seven studies, participants reported they felt little choice in this decision as they believed that sex work was the only way to survive financially—although two studies reported that a few individuals felt they did retain agency whilst engaging in sex work. Several studies described how sex work led to risky sexual behaviour with exposure to sexually transmitted infections (STIs) and human immunodeficiency virus (HIV) reported in South Africa, Bangladesh, and Uganda. Others referenced how sex work led to increased vulnerability and exposure to sexual assault. HIV was discussed in detail in Mauritania, citing stigma and financial difficulties as key pathways for Senegalese gay men to lead to disrupted HIV treatment, poor physical outcomes and even death [56].

One quantitative study examined risk factors and prevalence of HIV in refugees in Lebanon and found higher proportions of those engaged in sex work in displaced Syrians and displaced Iraqis compared to non-displaced Palestinians (62%, 42% and 4% respectively, *p* < 0.001). This study also found that higher proportions of displaced groups had unprotected anal sex with partners whose HIV status was positive or unknown (48% Palestinian, 58% Syrian, 64% Iraqi, *p* = 0.02) [59]. In Germany, those residing in the LGBTIQ shelter had 2.8 times the odds of having a chronic illness compared to those in non-LGBTIQ shelters (CI 1.15–7.13, *p* < 0.05) [53].

## 4. Discussion

This systematic review presents important evidence on forced migrants with diverse sexual orientation, gender identity or expression. Migration has become an increasing global response to conflict, persecution, violence and climate change [65,66,67], which has included those who are persecuted based on SOGIE [68,69]. It is important to understand the risks and protection needs that these forced migrants face during transit, which differ from heteronormative forced migrants [34]. Knowledge of protection needs will enable actors in humanitarian situations and along migration routes to respond to the serious gap in services appropriate to individuals with sexual and gender diversity [34].

**Figure 4:**
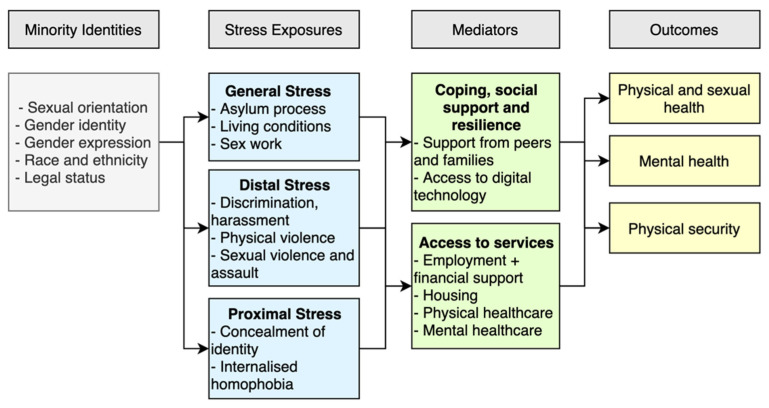
Framework describing potential pathways of minority stress amongst forced migrants with diverse SOGIE.

Figure 4 shows the conceptual framework developed based on the findings of this review, which has been designed to inform practice, policy and research. The framework is guided by the pathways of stress described by the minority stress model, but adapts it to apply to the situation of forced migration. The framework consists of four stages that shape health experiences during the migration journey and beyond, which include: (i) minority identities; (ii) stress exposures; (iii) mediators; and (iv) outcomes. These stages are modelled on the minority stress model, but we provide greater analysis based on the thematic findings of the study to examine pathways of stress in forced migrants with diverse SOGIE. The framework will require further research and investigation to build on the limited evidence provided here.

### 4.1. Conceptual Framework

#### 4.1.1. Minority Identities

Minority identities are comprised of an individual’s characteristics that often define their social position, often reflecting social hierarchy and privilege. Features of individuals’ social identity intersect to shape interpersonal interactions and are often the bases for marginalisation. This review focused on minority identities around sex and gender, however, the findings also highlighted race, ethnicity and legal status as individual characteristics that can further compound SOGIE challenges. Intersections of gender and race have been described in different contexts, this review analyses these characteristics using a migration lens.

#### 4.1.2. Stress Exposures

Stress exposures are defined as the additional stressors beyond minority status, such as environmental conditions or day-to-day behaviors and interactions with others. These exposures can be viewed as general, distal, and proximal stressors, indicating the proximity of the stress to the individual. For the purposes of this framework, these stressors are conceptualised separately, but in reality, they are likely to interact and overlap across categories.

General stress comprises those conditions in the context and individual circumstances that exacerbate stress among minority or marginalised groups, such as forced migrants. As indicated by the literature, difficulties with or rejection of asylum claims increases stress among migrants [70], particularly those in encamped settings or detention centers, which often degrades their mental health [71]. This review showed that diverse SOGIE migrants also experience these general stressors, and are likely to encounter greater stress than their migrant or local residential peers because of their minority status. The description of this category in the framework builds on the wider literature, however, it requires further research.

Distal stress is defined as experiences or actions that diverse SOGIE migrants experience from other individuals, which encompass discriminatory experiences during interpersonal exchanges. Exposures include, for example: accessing accommodation, employment, medical care, engaging in sex work, and sexual violence and assault. Our findings detail distal stressors SOGIE migrants experience from a variety of actors, including their compatriots. Research examining migration pathways for forced migrants in general has demonstrated vulnerabilities to abuse, and risk of physical and sexual abuse along transit routes [72,73,74]. Literature has also described victimisation experiences among SOGIE individuals before they migrate, particularly in countries with especially discriminatory gender norms, such as Uganda with severe homophobic and transphobic attitudes [2,33,49,50,55,60,61,75,76]. The findings related to this theme underscore the process and pervasiveness of discriminatory behaviors. The intersection of forced migrant identities and diversity of SOGIE likely results in dual marginalisation [38], and likely more negative health outcomes than non-SOGIE groups.

Proximal stress can be defined as the internal psychological processes that occur based on a minority individual’s actions or experiences, which are deeply influenced by their marginalisation. This review suggests that individuals concealed their gender identity as a short-term protection mechanism from harassment and to increase physical security. Concealment for protection is a feature consistent with research findings from a systematic review amongst LGBQ non-migrants [77]. Conversely, the literature reports negative outcomes from concealment, which the minority stress model reflects as a proximal stress process. Here, concealment of identity is conceptualised as a pathway to internalised homophobia and leads to shame and fear to disclose SOGIE, which research has corroborated [31,39,78]. Data from our review hint at links between concealment and social isolation, however there is not enough evidence to fully identify consequences of concealment in this population.

#### 4.1.3. Mediators

Mediators were identified as positive or negative factors that influence the outcomes. The findings provide examples of mediators, including coping, social support and resilience mechanisms, such as support from peers and families, and access to digital technology. The ability of individuals to access services, including, for example, physical and mental health care, employment opportunities, housing and financial support, are likely to have important mediating effects.

The literature on migrants during and after transit reports that religion, use of technology and social connections improve coping mechanisms and increase resilience [38]. For example, African migrants report the importance of communalism, both through familial ties and in developing new relationships with those from similar ethnic and cultural backgrounds, whilst on the move and in destination countries [79]. LGBTQI+ populations have found agency and resilience even within countries with highly restrictive gender laws, through building communities and support mechanisms and using digital technology and activism [80]. In the USA and Canada, studies on support structures for LGBTQI+ refugees highlight the importance of community, support groups, and access to healthcare, housing and employment [81]. From the findings in this review, these important coping strategies did not appear to be widely available to the SOGIE migrant population, particularly as a result of rejection by families and peers because of gender status. These results support the hypothesis that SOGIE forced migrants face additional challenges to their ability to manage stress and remain resilient when compared to other groups. It appears that digital media may be especially important to SOGIE migrants to connect to individuals in ways that are safe and to develop agency and grow confidence. This evidence can help identify the type of support that SOGIE migrants need to reduce exclusion and isolation.

Those with diverse SOGIE, and in particular transgender persons, have historically faced stigma and barriers accessing medical and mental health care [82]. The findings in this review, although limited, align with the literature that indicates that individuals denied sexual health services and appropriate mental health support, in particular. Hormone therapy has been shown to improve mental health outcomes and quality of life, but can be difficult to access even for those in post-migration context, and in those not on the move, with events such as COVID-19 reducing global access with knock-on consequences for mental health [83,84,85,86,87].

#### 4.1.4. Outcomes

Outcomes were defined as health of SOGIE migrants, particularly physical, sexual and mental health.

Research in non-migration and post-migration settings has indicated the mental health needs of LGBTQI+ populations [11,88,89], and emphasised the need for sensitive and appropriate care. Studies have highlighted intersections between poor mental health outcomes and minority status related to race, poverty and gender [90]. Furthermore, literature reviews on the mental health effects of trauma survivors from conflict settings have shown high prevalence of depression and anxiety [91]. Our findings share similarities with these reviews, particularly many similar features of poor mental health, such as nightmares, depressive symptoms, anxiety, loneliness and suicidal ideation. Several cross-sectional studies in this review provided a comparison with other populations (either non-migrant groups or heteronormative groups), and although the data were very limited, SOGIE migrants had higher likelihood of screening positive for mental illness than the comparison population. This finding is consistent with the hypothesis that excess stress and intersecting identities result in worse mental health outcomes. These findings require further quantification to explore directionality and causation, however they point towards a need for sensitive and timely access to mental health and psychosocial support services for SOGIE migrants during transit.

The findings also offered information on risks of HIV, STIs and sex work. Reports suggested that engagement in sex work arose from financial insecurity, which subsequently exposed these individuals to HIV infection, sexual violence and physical assault. Research has indicated that transgender non-migrants have high rates of HIV, especially amongst those who engage in sex work [92,93,94,95]. Research has also shown that transgender persons tend to be marginalised, left out of HIV care and not included in research, even despite awareness of the violence associated with this work [16]. A greater understanding and evidence about the pathways between sex work, HIV, and violence will be valuable for service providers to be able to intervene at key transit points to prevent abuse and illness. Assisting individuals to access to financial resources could help to prevent or reduce risk of harm.

### 4.2. Limitations

The paucity of data limits globally relevant inference. Lower quality evidence was included and given similar weight as higher quality studies. The majority of data were collected in locations that served multiple functions as separating phases of migration proved difficult. The articles identified did not cover all migration pathways: less research was identified from South Asia, Australasia and South America. More research is required to delineate nuances related to different settings. The review focused on transit experiences of migration due to limited resources, further research could examine the whole migration pathway. Furthermore, location-specific restrictions or local laws were not analysed due to the wide scope of the review. The restricted grey literature search meant specific routes or geographical contexts may have been missed. The review was restricted to English-language, as such there were limited studies based in Francophone Africa. Publication bias in the form of open-access data meant over twenty full-texts could not be accessed. Less data were available for lesbians, transgender women, non-binary or queer persons and transgender men, with over-reporting for gay men, and nuanced identities were not adequately identified. Furthermore, sampling frames in this review were based on individuals who already self-identified in public as ‘out’ or part of LGBTQI+ organisations, or who had disclosed their identity to authorities or NGOs. Experiences and outcomes of those whose SOGIE remains concealed are hidden. No literature specifically identified minors in their sample, therefore further possible vulnerabilities related to age could not be identified. A meta-analysis of quantitative data could not be performed due to heterogeneity in exposure, outcomes, and measurement tools.

The framework developed is based on the limited findings of the review and requires further research and investigation to build on the limited evidence revealed. It should be adapted for different contexts to those described here, and may not be appropriate for subsets of gender or sexual minorities.

### 4.3. Policy, Program and Research Implications

The findings of this review suggest the general neglect of SOGIE migrants in practice and in research and offer indications for improvements in policy, practice and research. And, perhaps most importantly, our findings strongly indicate the need to involve SOGIE individuals in future planning and decision-making to ensure proposed solutions prioritise their diverse protection and service needs.

The findings highlight that discrimination and abuse are common and significantly affect the health and safety of SOGIE groups. The literature also indicates the prevalent fear of unwanted or even voluntary disclosure. These results suggest that individuals may benefit from greater legal protection for forced migrants with diverse SOGIE combined with state-led anti-racist and anti-xenophobic messaging and measures to address intolerance of diverse SOGIE by other migrants, local residents, and particularly by authority figures.

Findings clearly indicate the hazards that occur during transit. Results suggest that SOGIE migrants might benefit from efforts by migration and rights organisations and governments to promote bilateral agreements and regulations related to safe passage, resettlement programs, and anti-discriminatory laws that specifically address the risks associated with diverse SOGIE. Given individuals’ risks and fears associated with this ‘limbo’ stage, SOGIE migrants would benefit from efficient processing of asylum and resettlement claims to minimise distress, and detention of SOGIE forced migrants, especially in transit situations where they are likely to be at substantial risk of harm.

Review results also highlight that many SOGIE migrants harbor substantial fears of having their status disclosed. Health and support service organisations are often well-positioned to implement safe environments for individuals to voluntarily disclose their status and access confidential gender-specific services. These types of processes can draw on existing guidance, which should be supplemented by input from diverse SOGIE representatives [36]. Furthermore, the literature indicates that access and use of services has been limited, which suggests the importance of sensitisation and training for staff from migration organisations to provide appropriate, customised support for SOGIE individuals. Training could include, for example, psychological first aid techniques, sensitive interview and communication approaches, and guidance on referral pathways into gender-specific mental and physical health care. Our review also identified training materials that have been developed for these purposes by UNHCR. However further resources should be developed for different settings, particularly those that include the participation and input from affected individuals.

Our findings further underline how seldom fully gender-disaggregated data are collected beyond normative binary male/female options. Yet, the results also suggest that requesting disclosure of SOGIE status can put individuals at risk. Therefore, SOGIE-informed options will need to be considered to ensure individuals disclose in ways they feel are safe and they receive the protection and care they need. SOGIE groups will need to be consulted to learn safe service and facility preferences, especially among transgender persons in camp and detention settings. Gender diversity should be taken into account when providing shelter to identify situations that offer individuals the greatest safety from abuse, and contexts that promote acceptance. Developing safe spaces that are tailored for gender non-conforming groups will require careful consideration of varying SOGIE group preferences, the gender norms and discrimination among other migrants and local residents, and knowledge and sensitivity of authorities who may be managing these facilities.

Simultaneously, the results suggested the dangers associated with the transit period, when individuals are likely to have little to no access to services or protection mechanisms. Organisations that manage migration can play a particularly important role in developing and identifying tailored interventions that enable diverse SOGIE groups to become aware of and get in touch with services in anonymous ways, if they wish. For example, in major transit locations, confidential contact points might be positioned to offer sensitised services, including mental health, sexual health services including HIV testing and treatment, and post-sexual assault services. Further, service providers should also be strongly encouraged to assist transgender persons access hormone treatment. Providing digital technology, such as mobile phone devices and internet or WiFi services, might also facilitate safe requests for services by forced migrants with diverse SOGIE.

Because of the substantial safety, discrimination and health risks, especially mental health concerns, associated with being a member of a diverse SOGIE group, our findings indicate how much more research is needed to learn how to address the various needs of the different members of this diverse group. Without any doubt, members of diverse SOGIE groups are the individuals who understand their risks and needs and, most importantly, the feasible options for possible disclosure, protection and care. Future research needs to involve all disaggregated gender groups, including non-binary and bisexual individuals, and transgender men and women. Gender terms should be culturally relevant and based on self-identification. Future research should also examine minors as a sub-group. Researchers should report on their biases and beliefs and utilise guidance available on ethical and sensitive practices [96,97]. Information should be gathered to understand further and quantify the consequences of the combination of minority identities, to examine compounded marginalisation effects, and further research using comparison population groups should be conducted.

## 5. Conclusions

Our review has highlighted the substantial safety risks and fears that forced migrants with diverse SOGIE often experience, frequently on a daily basis. Our results also indicate that, to date, their concerns have received extremely little attention in the migration discourse—and simultaneously, that migrant SOGIE have received limited recognition in LGBTQI+ conversations. By understanding pathways to poor versus good health outcomes for forced migrants with diverse SOGIE, there is substantial scope for actions to improve the situation of migrants worldwide. Findings from this review should be used to encourage greater recognition of the serious neglect and important concerns of SOGIE migrants, who need better support and acceptance be able to advocate for themselves while in transit.

## Figures and Tables

**Table 1:** Inclusion and exclusion criteria.

	Included	Excluded
Sample	People of all ages identifying as: (i) diverse SOGIE and (ii) refugees, asylum seekers, or forced irregular migrants Published after 1 January 2000 English language	Voluntary migrants Those with a traditional normative binary SOGIE Published prior to 1 January 2000 Non-English
Phenomenon of interest	Specific experiences during the migration journey that are related to SOGIE	Only pre-migration experiences
Design	Any study design that includes empirical evidence and not in exclusion list	Systematic reviews, reports, briefings, legal cases, commentaries, no empirical evidence
Evaluation	Mental and physical health during transit and immediately after arrival Services available and barriers to access	Integration experiences only
Research type	Peer-reviewed literature Grey literature Full text available	Full text not available

**Table 2:** Study Characteristics.

Author, Year	Objective	Setting	Study Design, Methods, Sampling	Study Population	Quality Appraisal Score
Alessi et al. 2018 [55]	To investigate how stress shapes migration experiences of LGBTQ refugees	Austria, Netherlands	Mixed methods: Cross-sectional survey, and qualitative interviews. Purposive sampling.	38 LGBTQ adults in Europe from MENA ^1^ + Asia. Asylum seekers: accepted, pending or rejected	100% CASP 80% AXIS High
Bayramoglu et al., 2018 [48]	To shed light on interaction of forced migration, sexuality, and media.	Germany	Qualitative ethnography, interviews and observation. Snowball sampling	10 adults: Syrian + Egyptian. Queer as defined by author. Refugees.	50% Medium
Bhagat et al., 2020 [49]	Explore queer forced displacement and how they survive and navigate spaces in Cape Town	South Africa	Qualitative interviews. Sampling unknown.	6 adults: Kenya, Zimbabwe, and DRC ^2^. Queer as defined by author. Refugees and asylum seekers	40% Medium
Broqua et al., 2021 [56]	To report on health and living conditions for Senegalese gay men in Mauritania applying for resettlement	Mauritania	Qualitative interviews. Snowball sampling	10 Senegalese men self-identified as gay. Refugees, asylum seekers, or rejected asylum seekers	30% Low
Clark et al., 2021 [57]	To ﻿assess and compare prevalence of psychiatric disorders among Lebanese and Syrian MSM and transgender women	Lebanon	Cross-sectional quantitative survey. Respondent driven, snowball sampling	488 MSM and transgender women: 230 displaced Syrians and 258 Lebanese. Forcibly displaced due to war or identity	65% Medium
Fox et al., 2020 [58]	To investigate how mental distress, social isolation, identity disclosure, and asylum procedures are interlinked for LGBTQ asylum seekers	US and Canada	Cross-sectional, quantitative online survey. Purposive sampling	308 Adults. LGBTQ refugees and asylum seekers	70% High
Golembe et al., 2020 [17]	To investigate how LGBTQ* refugees experience minority stress after migrating, including distal and proximal stress, and mental health	Germany	Qualitative FGDs ^3^, interviews, + demographic questionnaire. Convenience sampling	26 adults from MENA, Middle East, Asia. Self-identified as LGBTQ*. Refugees, attempted or rejected asylum seekers	100% High
Gottlieb et al., 2020 [53]	To provide insights into health and utilisation of services through comparison of LGBTIQ vs. non-LGBTIQ asylum seekers	Germany	Cross-sectional quantitative survey. Mix of non-random and weighted random sampling	32 responses from refugees and asylum seekers in LGBTIQ shelter.	85% High
Jafari F, 2014 [50]	To explore the impacts of race and sexual orientation on Iranian queer migrants	Turkey	Ethnographic+ qualitative interviews + participant observationUnknown sampling	19 adult queer Iranian men. Asylum seekers or refugees	30% Low
Pincock, 2020 [47]	To explore protection issues in communities among refugees in urban, camp, and settlement contexts in East Africa.	Kenya	Ethnographic + qualitative participant observation + interviews.Snowball sampling	LGBTI refugees. No sample size given	40% Medium
Tohme et al., 2016 [59]	To determine prevalence of HIV and psychosocial correlates among Iraqi, Syrian, and Palestinian MSM refugees in Lebanon	Lebanon	Quantitative cross-sectional surveys Long chain peer referral method sampling	150 adult refugees. Gay, bisexual, MSM	70% High
Amnesty International, 2017 [60]	To document the path of flight from Honduras, Salvador and Guatemala to Mexico and the US	Mexico	Qualitative interviews. Sampling unknown	20 refugees and asylum seekers identifying as gay men or trans women	40% Medium
Chynoweth S, Women’s Refugee Commission, 2019 [51]	To examine sexual violence experiences by refugee men and boys traveling the central Mediterranean migration route	Italy	Qualitative. Key informant interviews + FGDs. Purposive sampling	15 refugees and migrants with diverse SOGIESC ^4^.	90% High
Chynoweth S, Women’s Refugee Commission, 2019 [52]	To examine sexual violence experienced by refugees with diverse SOGIESC and trans women in Kenya	Kenya	Qualitative interviews + FGDs. Purposive sampling.	4 FGDs with refugees from the DRC, Somalia, and South Sudan with diverse SOGIESC	90% High
Dwyer E, The Edge Effect, 2021 [33]	Reports on violence and exclusion experienced by people with diverse SOGIESC in humanitarian settings	Bangladesh	Qualitative, interviews Sampling unknown	35 respondent: refugees with diverse SOGIESC	60% Medium
Ghosal N, Human Rights Watch, 2020 [61]	To document violence and discrimination against LGBT asylum seekers in the US and Central America	El Salvador, Guatemala, Honduras, Mexico and US	Qualitative interviews Sampling unknown	20 LGBT asylum seekers and refugees	60% Medium
ORAM, 2013 [62]	To explore protection issues amongst SGN ^5^ refugees in Mexico	Mexico	Qualitative, Interviews. Likely purposive sampling.	14 SGN refugees and asylum seekers	60% Medium
ORAM, 2013 [63]	To identify protection issues for LGBTI asylum seekers and refugees in South Africa	Cape Town	Qualitative, interviews Purposive sampling	14 SGN refugees and asylum seekers from Africa + Asia	60% Medium
ORAM, 2011 [64]	To document protection gaps facing LGBT refugees in Turkey	Turkey	Qualitative, interviews Purposive sampling	62 LGBT asylum seekers and refugees	60% Medium
ORAM, Refugee Law Project, 2013 [54]	To explore protection issues for SGN refugees in Uganda	Uganda	Qualitative, interviews Purposive sampling	25 SGN refugees from Africa	80% High

^1^ Middle East and North Africa. ^2^ Democratic Republic of Congo. ^3^ Focus group discussions. ^4^ Sexual orientation, gender identity, expression and sexual characteristics. ^5^ Sexual and gender nonconforming. N.B. asterisk is used as truncation.
